# Fruit volatilome profiling through GC × GC-ToF-MS and gene expression analyses reveal differences amongst peach cultivars in their response to cold storage

**DOI:** 10.1038/s41598-020-75322-z

**Published:** 2020-10-27

**Authors:** Antonella Muto, Carsten T. Müller, Leonardo Bruno, Laura McGregor, Antonio Ferrante, Adriana Ada Ceverista Chiappetta, Maria Beatrice Bitonti, Hilary J. Rogers, Natasha Damiana Spadafora

**Affiliations:** 1 Department of Biology, Ecology and Earth Sciences, University of Calabria, Via Ponte P. Bucci 6b, 87036 Arcavacata Di Rende, Cosenza, Italy; 2 School of Biosciences, Cardiff University, Sir Martin Evans Building, Museum Avenue, Cardiff, CF10 3AT UK; 3 SepSolve Analytical Ltd, Swan Court, Hampton, PE7 8GX Peterborough UK; 4 Department of Agricultural and Environmental Science—Production, Landscape, Agroenergy, Università Degli Studi Di Milano, Via Celoria 2, 20133 Milan, Italy; 5 Markes International, Gwaun Elai Medi-Science Campus, Llantrisant, Cardiff, UK

**Keywords:** Plant biotechnology, Agricultural genetics

## Abstract

Peaches have a short shelf life and require chilling during storage and transport. Peach aroma is important for consumer preference and determined by underlying metabolic pathways and gene expression. Differences in aroma (profiles of volatile organic compounds, VOCs) have been widely reported across cultivars and in response to cold storage. However, few studies used intact peaches, or used equilibrium sampling methods subject to saturation. We analysed VOC profiles using TD-GC × GC-ToF-MS and expression of 12 key VOC pathway genes of intact fruit from six cultivars (three peaches, three nectarines) before and after storage at 1 °C for 7 days including 36 h shelf life storage at 20 °C. Two dimensional GC (GC × GC) significantly enhances discrimination of thermal desorption gas chromatography time-of-flight mass spectrometry (TD-GC-ToF-MS) and detected a total of 115 VOCs. A subset of 15 VOCs from analysis with Random Forest discriminated between cultivars. Another 16 VOCs correlated strongly with expression profiles of eleven key genes in the lipoxygenase pathway, and both expression profiles and VOCs discriminated amongst cultivars, peach versus nectarines and between treatments. The cultivar-specific response to cold storage underlines the need to understand more fully the genetic basis for VOC changes across cultivars.

## Introduction

The peach (*Prunus persica* (L.) Batsch) is a fruit tree belonging to the *Rosaceae* family and is one of the most commercially important species of this clade, including a wide range of varieties and cultivars^1,2^. Peach fruits are widely consumed, being greatly appreciated for their distinctive flavour, texture, juiciness, and nutritional value. Among the parameters that define peach quality their aroma is one of the most important from the consumers’ point of view.

Fruit flavour is determined by volatile organic compounds (VOCs) together with sugars and acids, which accumulate during fruit development and ripening. While the VOC profile determines fruit aroma, the latter contribute to sweetness and tartness^3^. However, VOCs seem to determine flavour differences the most, across different peach cultivars and groups^4^. Around 100 compounds have been identified in the skin and flesh of peaches^6–8^, and among them C6 compounds, esters, benzaldehyde, linalool, C13 norisoprenoids and lactones were the most abundant. Of these, about 30 determine the typical peach aroma, in particular, lactones, above all γ- and δ-decalactone, have been reported to act in association with C6 aldehydes, aliphatic alcohols, such as n-hexanal, (E)-2-hexenal, and (E)-2-hexenol, esters, such as (Z)-3-hexenyl acetate, norisoprenoids, phenylpropanoids and terpenes^4,6,8,9^. Differences in aroma profiles have been noted both amongst individual cultivars^8^ and across different peach varieties such as honey peach, flat peach and nectarine^4,10^. For example, Wang et al*.*^10^ identified four groups of VOC profiles with characteristic patterns of abundance of different VOC families across 50 cultivars of peaches.

VOCs, can be strongly affected by post-harvest treatment and storage conditions^4,12–15^. Post-harvest storage is unavoidable for fruit shipment to the market, and the storage period between fruit harvest and purchase by consumers can be quite long, especially when fruits are exported to distant markets. This period can comprise 3–5 weeks including shipping, handling at production, and receipt^16^. Peach fruits are characterized by a rapid deterioration at room temperature^17,18^ and therefore cold storage (0–1 °C) is commonly applied as the most suitable method for delaying ripening after harvest and extending fruit commercial life^19,20^. However, as in many fruit species, low temperature storage can lead to chilling injury (CI), a collective term for several visible physiological disorders that affect fruit quality^21^. These include changes in texture but also invisible damage including loss of flavour and a decline in aroma quality^18,21^. Resilience to post-harvest storage varies across peach cultivars^22^: for example, generally nectarines have better storage characteristics than peaches^23^. Both visible and invisible cold damage have a commercial impact because they are not detectable until the fruit reaches the consumers. Hence, understanding the overall mechanisms by which peach fruits respond to cold stress is of considerable economic and scientific interest.

Despite significant progress in understanding the molecular and genetic basis of whole plant acclimation to cold stress, fruit biology research in this area has lagged behind and has mainly focussed on fruit flavour^13,24^. In general, aldehydes tend to decline, while esters increase in the fruit, and post-harvest treatments, such as low temperature and controlled atmosphere, have been used to investigate post-harvest changes in peach aroma quality^24,25^. In particular, cold storage was found to induce the production of alcohols and aldehydes, with esters and lactones increasing following shelf life storage at 20 ºC after the cold treatment. Moreover, differences in metabolite and VOC profiles amongst different peach cultivars subjected to cold storage has also been explored^12^. Amongst cultivars, there were differences in the relative abundance of alcohols, C6 VOCs and esters, while aldehyde VOC composition was less related to cultivar.

In the last decade, progress has been also made in understanding the biosynthetic pathways of fruit flavour chemicals and their regulation, and some of the genes involved in these pathways have also been identified^26–28^. Many of the VOCs identified from peach aroma derive from polyunsaturated fatty acids (PUFAs)^29,30^. The first step of fatty acid degradation for ester biosynthesis is catalysed by LOX enzymes^31^. LOX enzymes can be grouped into two categories based on the specificity of the position in which they oxygenate fatty acids: at C9 (9-LOX) or C13 (13-LOX) of the hydrocarbon skeleton or the presence/absence of a transit peptide^32^. Peach genes *PpLOX2, PpLOX3, and PpLOX4*, cluster in the 9-LOX class, while *PpLOX1* is a 13-LOX^33^. Moreover, in peach, *PpLOX1* and *PpLOX4* were also associated with the synthesis of lactones, and *PpLOX2* and *PpLOX3* with the synthesis of C6 aldehydes^33,34^.

Other key enzymes of the LOX pathway, including hydroperoxide lyase (HPL), alcohol deydrogenase (ADH) and alcohol acyl transferase (AAT) have been identified from peach^24,30,33^. Fatty acid desaturases, *FAD* genes, are required for the biosynthesis of polyunsaturated fatty acid^35^ and also play a key role in the modification of the fluidity of lipid membranes, which is characteristic of species that respond to cold^36,37^. In tomato, altering fatty acid composition by overexpression of *FAD* genes both influenced CI tolerance and modified fruit volatiles^38^. The role of plant FADs associated with volatile formation has not been extensively studied although in peach, *ppFAD1* seems to be involved in the production of a precursor of lactones/esters^39^.

VOCs derive from oxidative cleavage of hydroperoxy fatty acids, the primary products of the LOX pathway, leading to the formation of short chain C6 or C9 volatile aldehydes and the corresponding C12 or C9 ω-fatty acids^40^ by action of HPL enzymes. C6 aldehydes are then reduced via alcohol dehydrogenases (ADH), leading to the formation of corresponding C6 alcohols^29^. Esters, which are the main group of VOCs in most fruits^25,41^, derive from the action of acyl alcohol transferases (AAT) which use alcohols as substrates to generate esters^32^. Lactone VOCs derive from a different pathway, through the hydrolysis of the epoxide group of fatty acids^42^ catalysed by epoxide hydrolase (EPH). The wide range of terpenes found in fruit VOCs are catalyzed by a large family of terpene synthases (TPS)^43^ some of which have been identified in peach. In peach fruits, some terpenes such as linalool, impart a sweet, floral and alcoholic note that is important for flavour quality^6,9^. Moreover, recently *PpTPS1* was localized to plastids and associated with the production of linalool, while *PpTPS2* is responsible for (*E*,*E*)-α-farnesene biosynthesis in the cytoplasm^44^. Notably peach fruit-specific transcriptional control of cold responses has recently been reported and linked to metabolite changes related to chilling injury^17^.

A key factor influencing both the number and the specific VOCs detected in peach aroma is the method of analysis. Early studies^8,45^ used steam distillation and hexane extraction from frozen homogenized samples. More recently solid phase microextraction (SPME) has been widely used, however many studies still report profiles from homogenized tissue^10,30,46^. Moreover, SPME is based on an equilibrium process, making quantitative analyses of VOC profile changes more challenging, and can suffer from saturation. In our work on fruit aroma^47^ we have favoured the use of thermal desorption tubes for capturing VOCs directly from whole or minimally processed samples, and in peach this has also proven to be feasible^48^ revealing important changes in VOC composition through storage.

Here data are presented comparing both VOC profiles and expression of VOC-related genes across three peach and three nectarine cultivars grown under the same environmental conditions before and after storage, using an enhancement of thermal desorption gas chromatography time of flight mass spectrometry (TD-GC-ToF-MS), namely TD-GC × GC-ToF-MS. Improved chromatographic resolution is provided by coupling two GC columns of different selectivity. Thus, compounds with similar retention times in the first dimension can be separated in the second dimension based on a different chemical property. To the authors’ knowledge this is the first time this technique has been applied to the analysis of peach aroma. Results reveal significant differences across cultivars indicating the importance of considering both aroma and the effects of cold storage in future breeding programmes, traits that are currently often neglected.

## Results

### VOC bouquet and changes in the abundance of VOC families across cultivars after cold storage

The following cultivars were analysed: ‘Sagittaria’, ‘Rome Star’ and ‘Summer Rich’ peaches and ‘Big Bang’, ‘Big Top’ and ‘Carene’ nectarines, and a total of 115 VOCs were putatively identified from all six cultivars by retention index and spectral comparison against the NIST library (Supplementary Table S1a,b online). These included nine acetate esters and 27 non acetate esters, 15 alkanes, 16 terpenes, 12 ketones, 11 cycloalkanes, six alcohols, five lactones, five aldehydes, four aromatic compounds, four alkenes, and one alkyne. Thermal desorption coupled with GC × GC-ToF MS analysis enabled high-sensitivity sampling and separation of a wide range of VOCs, allowing a comprehensive chemical signature to be obtained for each cultivar. The more polar components, aldehydes, and esters, were well-resolved from the less polar terpenes and n-alkanes, resulting in clear spectra and confident identification. For example, C33 (*cis*-3-hexenyl acetate) and C34 (*α*-phellandrene) share the same retention time on the first dimension of the GC × GC (13.88 min) while they are clearly separable on the second dimension (Supplementary Fig . S1 online).

Across all samples, terpenes, showed the highest mean relative abundance, significantly higher than any of the other VOC families apart from alkanes (*P* = 1.0), and non-acetate esters (*P* = 1.0) (Kruskal Wallis, *P* = 0, Dunn post hoc test, *P* = 0.0002 for lactones and acetate esters, *P* = 0 for all others). In some cultivars relative abundance of VOC families also varied between the timepoints (Table 1). However, there was no significant difference in VOC families amongst cultivars or between storage times. Differences in relative abundance of aldehydes and aromatic VOCs were more significant amongst cultivars after cold storage than before, whereas the opposite was the case for ketones. In five cultivars there was a significant difference in the relative abundance of a VOC family after storage compared to before storage (Table 1): acetate esters in ‘Carene’ (*P* = 0.016), aldehydes and ketones in ‘Sagittaria’ (*P* = 0.045 and *P* = 0.0027), alkanes in ‘Rome Star’ (*P* = 0.019), and the aromatic VOCs in ‘Big Bang’ (*P* = 0.003) (student t-tests, Table 1). Across all the cultivars, the relative abundance (Kruskal Wallis followed by a Dunn’s test) of non-acetate esters (*P* = 0.0004), aldehydes (*P* = 0.0140) and lactones (*P* = 0.0245) increased significantly while alkanes (*P* = 0.02) and alkenes (*P* = 0.023) decreased significantly after cold storage (Table 2). In contrast, abundance of other VOC families (acetate esters, alcohols, aromatic VOCs, cycloalkanes, ketones, terpenes and the alkyne) was not significantly affected by storage. For six VOC families relative abundance varied between peaches and nectarines (Kruskal Wallis followed by a Dunn’s test): acetate esters (*P* = 0), and lactones (*P* = 0.012) were more abundant in nectarines, whereas alcohols (*P* = 0.019), alkanes (*P* = 0.033), and terpenes (*P* = 0.031) were higher in peaches. (Table 2).

**Table 1:** Relative abundance of VOC families across the six peach varieties after 36 h at 22 ˚C following 0 or 7 days of cold storage.

VOC classes	DAY 0											
	S (P)^a^		BB (N)		C (N)		BT (N)		SR (P)		RS (P)	
Acetate ester	5.81 ± 3.93	ab	3.02 ± 3.63	ab	0.00 ± 0.00*	a	0.00 ± 0.00	a	14.08 ± 5.72	b	9.91 ± 8.04	ab
Non-acetate ester	8.66 ± 3.93	ab	6.40 ± 3.63	ab	14.03 ± 0.00	ab	8.40 ± 0.00	ab	22.92 ± 5.72	b	2.70 ± 8.04	a
Alcohol	0.93 ± 0.87	ns	4.55 ± 3.24	ns	5.09 ± 6.03	ns	2.14 ± 1.98	ns	0.00 ± 0.00	ns	0.00 ± 0.00	ns
Aldehyde	0.00 ± 0.00*	ns	0.00 ± 0.00	ns	0.86 ± 0.74	ns	0.00 ± 0.00	ns	2.51 ± 2.21	ns	0.00 ± 0.00	ns
Alkane	29.38 ± 19.72	ab	9.66 ± 2.01	b	51.59 ± 9.21	a	24.30 ± 19.86	ab	11.19 ± 8.10	b	23.36 ± 5.64*	ab
Alkene	9.08 ± 12.26	ns	0.06 ± 0.06	ns	0.00 ± 0.00	ns	0.00 ± 0.00	ns	0.00 ± 0.00	ns	1.66 ± 1.47	ns
Alkyne	0.00 ± 0.00	ns	0.00 ± 0.00	ns	0.00 ± 0.00	ns	0.00 ± 0.00	ns	0.80 ± 0.77*	ns	0.00 ± 0.00	ns
Aromatic	2.48 ± 1.84	ns	0.00 ± 0.00*	ns	1.90 ± 2.29	ns	0.00 ± 0.00	ns	0.92 ± 0.79	ns	0.83 ± 0.73	ns
Cycloalkane	1.28 ± 0.90	ns	2.63 ± 2.07	ns	7.89 ± 3.08	ns	27.06 ± 21.48	ns	1.39 ± 1.05	ns	1.97 ± 0.68	ns
Ketone	0.69 ± 0.65*	ab	0.01 ± 0.00	b	9.64 ± 2.95	a	2.32 ± 2.01	ab	4.51 ± 4.30	ab	7.00 ± 10.65	ab
Lactone	2.33 ± 1.22	ab	7.41 ± 12.51	ab	0.00 ± 0.00	b	0.00 ± 0.00	b	5.66 ± 3.45	ab	22.80 ± 10.55	a
Terpene	35.94 ± 18.47	bc	66.54 ± 8.42	a	9.06 ± 6.53	c	35.86 ± 6.61	bc	36.09 ± 4.89	b	29.83 ± 7.70	bc

VOC classes	DAY 7											
	S (P)		BB (N)		C (N)		BT (N)		SR (P)		RS (P)	
Acetate ester	14.19 ± 1.36	ab	4.41 ± 4.22	ab	1.96 ± 0.43	ab	1.94 ± 2.05	a	24.37 ± 3.31	b	5.28 ± 5.28	ab
Non-acetate ester	47.35 ± 1.36	ns	15.89 ± 4.22	ns	13.96 ± 0.43	ns	19.76 ± 2.05	ns	23.95 ± 3.31	ns	27.43 ± 5.28	ns
Alcohol	3.22 ± 2.14	ns	6.86 ± 5.18	ns	0.70 ± 1.09	ns	1.95 ± 2.27	ns	0.01 ± 0.00	ns	21.78 ± 24.19	ns
Aldehyde	12.86 ± 4.88	a	0.00 ± 0.00	b	0.74 ± 0.70	ab	2.22 ± 1.92	ab	3.11 ± 1.59	ab	0.00 ± 0.00	b
Alkane	8.29 ± 1.28	ab	11.42 ± 4.96	ab	49.39 ± 6.36	b	11.25 ± 8.83	ab	9.55 ± 3.87	ab	0.37 ± 0.31	a
Alkene	0.00 ± 0.00	ns	0.15 ± 0.14	ns	0.00 ± 0.00	ns	0.00 ± 0.00	ns	0.00 ± 0.00	ns	0.00 ± 0.00	ns
Alkyne	0.00 ± 0.00	ns	0.00 ± 0.00	ns	0.00 ± 0.00	ns	0.00 ± 0.00	ns	0.00 ± 0.00	ns	0.00 ± 0.00	ns
Aromatic	5.16 ± 3.58	a	0.59 ± 0.06	ab	0.26 ± 0.23	ab	2.21 ± 1.00	ab	0.00 ± 0.00	b	0.00 ± 0.00	b
Cycloalkane	1.11 ± 0.55	ns	3.12 ± 3.01	ns	1.67 ± 0.59	ns	2.00 ± 1.92	ns	4.24 ± 1.75	ns	4.19 ± 4.64	ns
Ketone	6.68 ± 1.08	ns	0.39 ± 0.43	ns	4.74 ± 4.16	ns	5.37 ± 6.58	ns	4.90 ± 1.18	ns	3.95 ± 5.28	ns
Lactone	1.75 ± 0.79	b	3.39 ± 1.99	b	14.96 ± 7.15	a	4.77 ± 2.31	b	11.58 ± 1.36	ab	10.92 ± 3.95	ab
Terpene	9.34 ± 5.11	c	52.46 ± 11.46	a	11.14 ± 4.89	c	48.62 ± 9.25	ab	32.82 ± 12.59	abc	24.28 ± 9.66	bc

^a^S = Sagittaria, BB = Big Bang, C = Carene, BT = Big Top, SR = Summer Rich, RS = Rome Star, P = Peach, N = Nectarine, mean ± SE; different letters and * indicate statistically significant differences amongst cultivars and time of storage respectively (ANOVA followed by Tukey’s post hoc test or Kruskal Wallis followed by Dunns post hoc test *P* < 0.05, depending on Shapiro–Wilk test for normality and Fligner–Killeen test for homogeneity of variances, n = 3), ns indicates no significant difference across all cultivars.

**Table 2:** Differences in total relative abundance of VOC families in relation to day of storage and peach versus nectarine.

	Acetate esters	Non-acetate esters	Alcohols	Aldehydes	Alkanes	Alkenes	Alkyne	Aromatic VOCs	Cyclo alkanes	Ketones	Lactones	Terpenes
Mean total relative abundance (TRA)	7.08	17.62	1.56	1.86	19.98	0.92	0.07	1.2	4.88	4.18	7.13	32.67
± SD	7.76	14.47	1.62	3.82	17.81	3.89	0.29	1.89	8.88	4.64	8.05	18.98
Mean TRA D0	5.47	10.52	1.19	0.56	24.91	1.8	0.13	1.02	7.04	4.03	6.37	35.56
Mean TRA D7	8.69	24.72	1.94	3.16	15.04	0.03	0	1.37	2.72	4.34	7.89	29.78
*P* value*	ns	0	ns	0.01	0.02	0.02	ns	ns	ns	ns	0.03	ns
Mean TRA for peaches (S, SR, RS)	12.27	22.17	1.33	3.08	13.69	1.79	0.13	1.57	2.37	4.62	9.17	28.05
Mean TRA for nectarines (BB, C, BT)	1.89	13.07	1.79	0.64	26.27	0.04	0	0.83	7.4	3.74	5.09	37.28
*P* value*	0	ns	0.02	ns	0.03	ns	ns	ns	ns	0.15	0.01	0.03

*Based on ANOVA followed by a Tukey’s test, or a Kruskal Wallis followed by a Dunn’s test if the data did not conform to the normality and equal variance required for a parametric test.

### Discrimination of VOC profiles amongst cultivars before and after cold storage

The overall pattern of relative abundance of VOCs varied strongly between samples and 44% of the variability was accounted for by the variables (PerMANOVA). Nonetheless, significant differences between profiles were found amongst cultivars (PerMANOVA, *P* < 0.001, R^2^ = 0.18), before and after cold treatment (PerMANOVA, *P* < 0.001, R^2^ = 0.06), and between nectarine and peach cultivars (PerMANOVA, *P* < 0.001, R^2^ = 0.07). There was a significant interaction between cultivar and cold treatment (PerMANOVA, *P* < 0.001, R^2^ = 0.13). Overall, the PerMANOVA analysis accounted for 44% of the variation of the data set.

**Figure 1:**
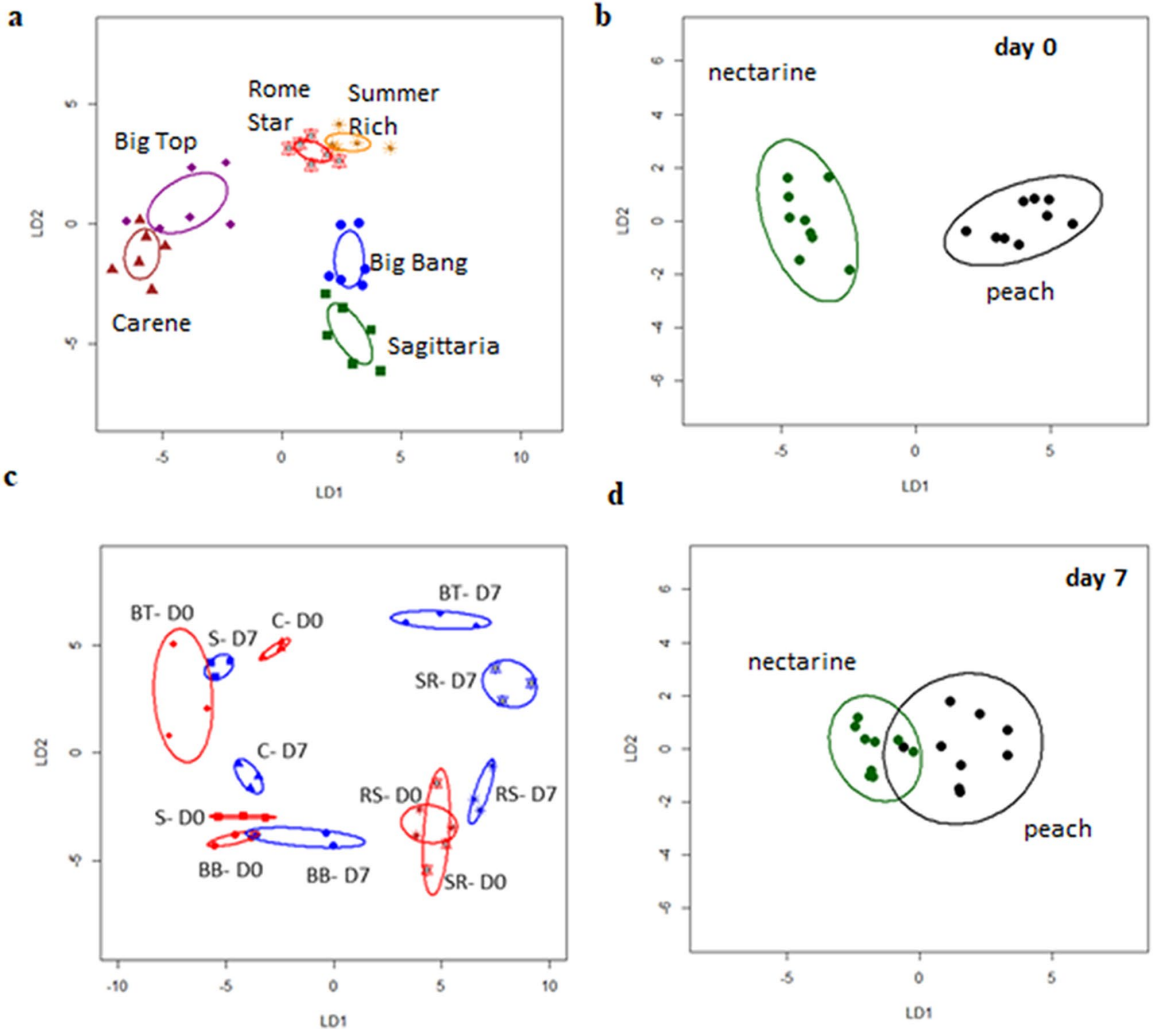
Linear discriminant (LD) plots from Canonical Analysis of Principal coordinates (CAP) based on all VOCs analysed using TD-GC-TOF-MS from six peach cultivars: ‘Sagittaria’ (S), ‘Big Bang’ (B), ‘Big Top’ (BT), ‘Carene’ (C), ‘Rome star’ (RS) and ‘Summer Rich’ (SR), by: (**a**) cultivar, (**b**) and (**d**) considering nectarine versus peach cultivars before (**b**) and after (**d**) storage; (**c**) cultivar analysed as a single category before (D0, in red) and after (D7, in blue) storage; storage was for 7 days at 1 ℃ followed by a 36 h recovery at 20 ℃. Each ellipse represents the 95% confidence interval. The plots use LD1 (**a**) with a percentage of correct classification = 91.7% (*P* < 0.0001; n = 6;  ± SE; (**b**) 100% (*P* = 0.01; n = 9; ± SD); (**c**) 91.7% (*P* < 0.0001; n = 3; ± SD); (**d**) 88.9% (*P* = 0.03; n = 9 ; ± SD).

Linear discrimination (LD) plots produced from CAP analysis separated each cultivar from all the others (*P* < 0.05), with a percentage of correct classification of 91.7% (*P* < 0.0001) (Fig. 1a). However, taking all cultivars together VOC profiles were not able to discriminate between the fruit before and after cold storage (Supplementary Fig. S2a online). Linear discriminant plots based on CAP analysis indicated VOC profiles were also not distinct between nectarines versus peaches when both time points of all cultivars were considered together (Supplementary Fig. S2b online). However, VOCs were able to discriminate between nectarines and peaches before, but not after, cold storage (Fig. 1b, d).

CAP analysis performed on cultivars and storage time combined into a single category (12 samples) resulted in correct classification of 91.7% (*P* < 0.0001) (Fig. 1c). VOC profiles discriminated between all cultivars before cold storage except for cv.s ‘Rome Star’ and ‘Summer Rich’. After cold storage there was complete discrimination across all the cultivars. However, cold treated ‘Sagittaria’ was not discriminated from the ‘Big Top’ VOC profile without cold storage. Within each cultivar VOC profiles before and after cold storage were distinct apart from ‘Big Bang’.

### Identification of volatile signatures to discriminate cultivar, cold treatment, and between nectarines versus peaches

**Figure 2:**
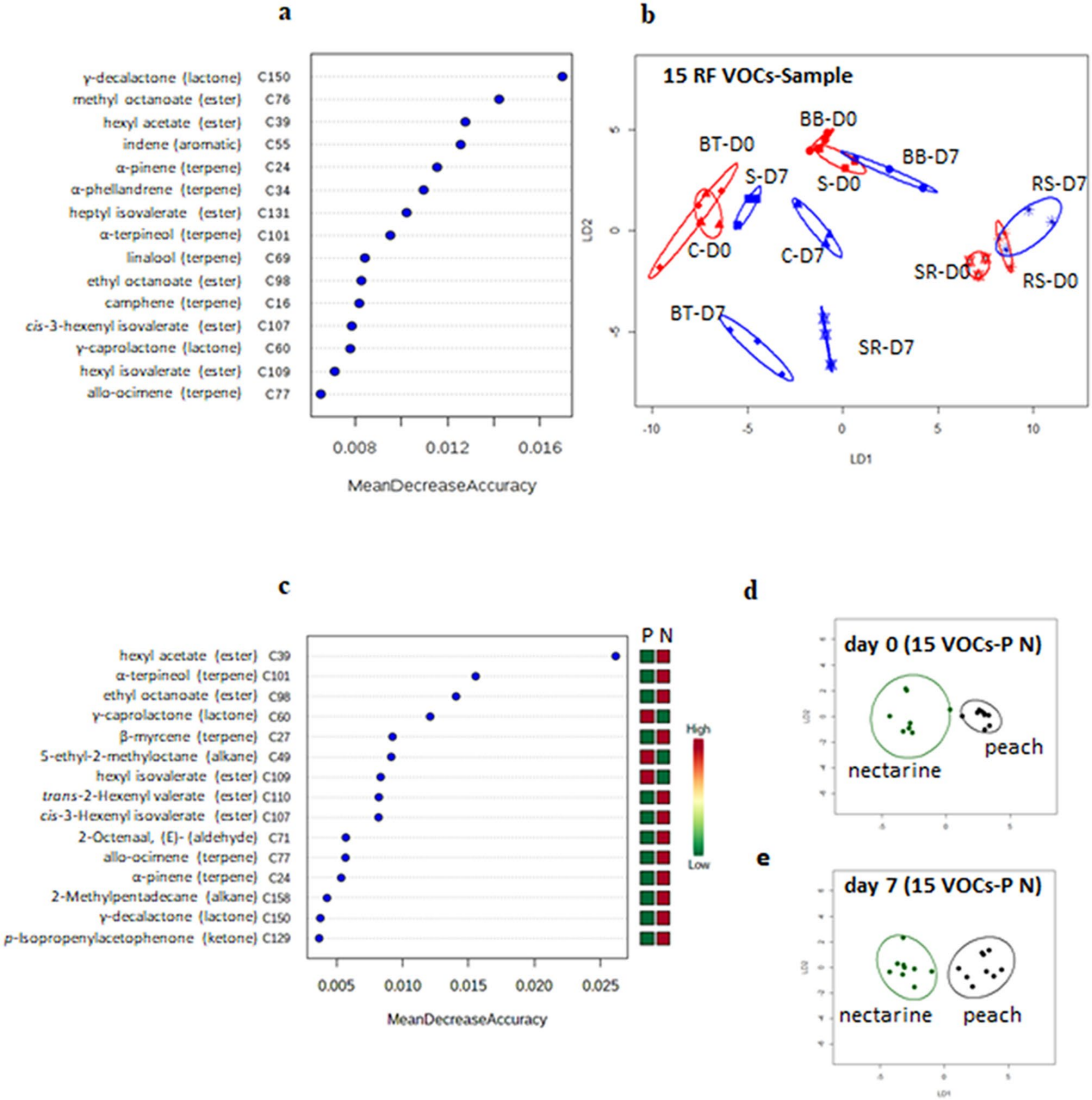
Mean decrease accuracy (**a**, **c**) and CAP analysis (**b**, **d**) for the 15 most significant VOCs identified by Random Forest (RF) analysis within Metaboanalyst contributing to discrimination between (**a**, **b**) VOC profile across all cultivars and timepoints (**c**, **d**, **e**) nectarine (BB, C, BT) versus peach (S, SR, RS) cultivars. For the CAP analysis each ellipse represents the 95% confidence interval of SD. The plots use LD1 and LD2 with a percentage of correct classification (**b**) 75% (*P* = 1; n = 3; ± SD); (**d**) 94% (*P* = 0.02; n = 9; ± SD); (**e**) 89% (*P* = 0.01; n = 9; ± SD). In the heat map peach association is denoted by “P” and nectarine by “N”.

Fifteen top ranked VOCs were identified using Random Forest (RF) for discrimination across all the samples (Fig. 2a) comprising six terpenes, six esters, two lactones and one aromatic VOC. Using the 15 VOCs identified by RF in relation to sample resulted in a similar discrimination compared to the full VOC set (PerMANOVA, *P* < 0.0001, R^2^ = 0.74 compared to *P* < 0.0001, R^2^ = 0.44 for the full dataset). A linear discriminant plot following CAP showed that there was some reduction in discrimination, although the majority of the profiles for the individual cultivars, apart from ‘Rome Star’ were discriminated between day 0 and day 7 of storage with a correct classification of 75% (Fig. 2b). When RF was applied to the VOC data considering only discrimination into peach and nectarine cultivars, the 15 VOCs showing most discrimination included four terpenes that were positively associated with nectarine, five esters four of which were positively associated with nectarine and one with peach, two lactones and two alkanes that showed contrasting association, and one alkane that was positively associated to peach (Fig. 2c). Linear discrimination plots of CAP analysis clearly discriminated the VOCs between the two groups, peaches and nectarines both before and after cold storage (Fig. 2d, e).

### Expression patterns of genes related to VOCs are modulated by cultivar, cold treatment, and peach versus nectarine type

**Figure 3:**
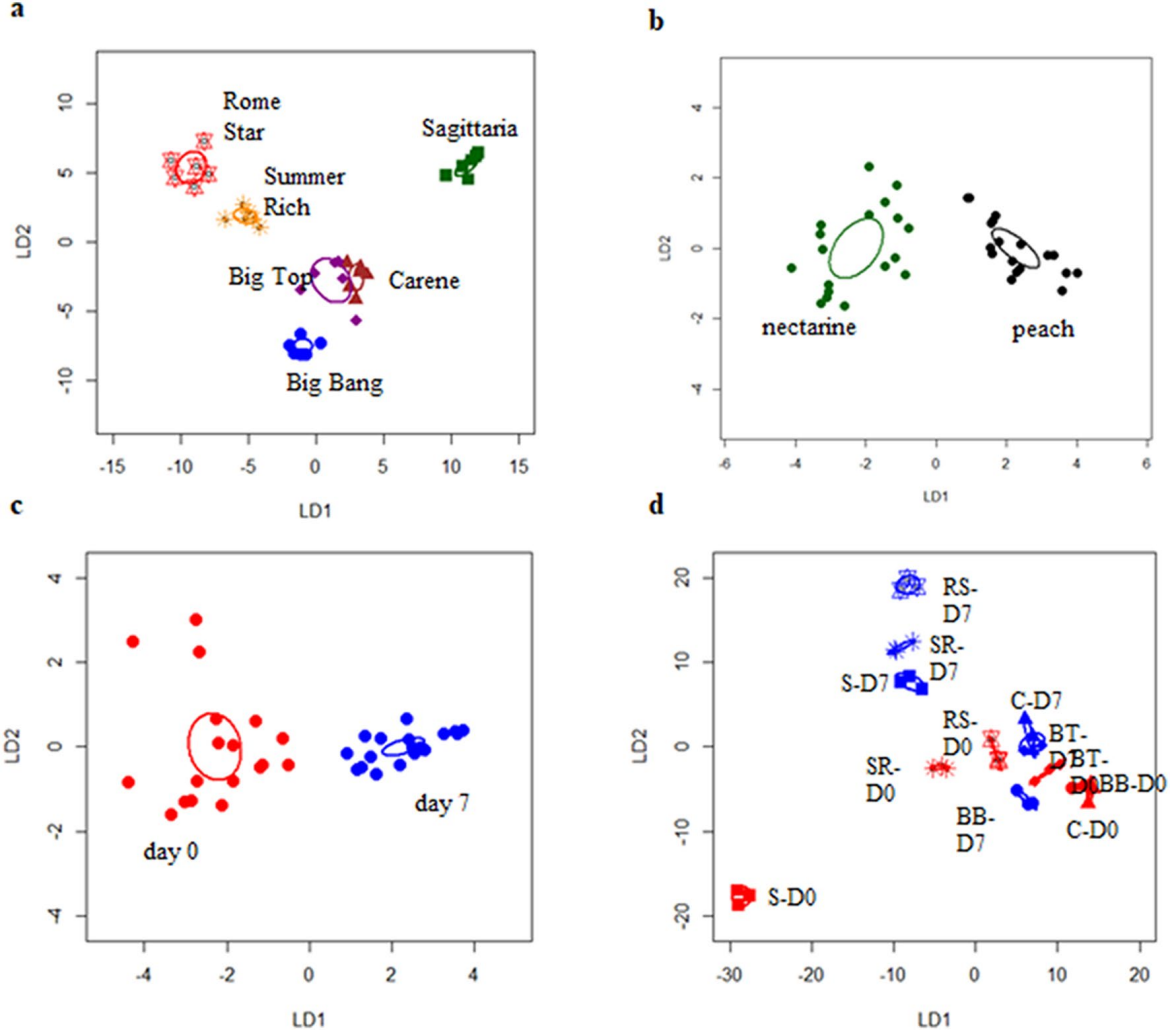
Linear discriminant plots from Canonical Analysis of Principal coordinates (CAP) based on all gene expression from six peach cultivars: ‘Sagittaria’, ‘Big Bang’, ‘Big Top’,‘Carene’, ‘Rome star’ and ‘Summer Rich’, by (**a**) cultivar, (**b**) peaches versus nectarines, (**c**) time of storage, (**d**) the six peach cultivars analysed as individual category before (D0, in red) and after a 7 day (D7, in blue) cold. (mean ± SE). Each ellipse represents the 95% confidence interval of SE. The plots use LD1 with a percentage of correct classification (**a**–**d**) = 100% (*P* < 0.01; and n = 6; n = 18; n = 18; n = 3 respectively).

The combined expression pattern of all the analysed genes was able to discriminate cultivars, time of storage and peaches versus nectarines, and there was also a significant interaction of time and cultivar using PerMANOVA (cultivar R^2^ = 0.54 , time R^2^ = 0.11, , peach versus nectarine R^2^ = 0.16 and interaction R^2^ = 0.09 , all *P* < 0.01). CAP allowed 100% correct identification of cultivars (Fig. 3a), and peach versus nectarine (Fig. 3b) and time of storage (Fig. 3c). Although a linear discriminant plot of the CAP analysis also separated most samples (cultivar x time) (Fig. 3d), there was a lack of discrimination in the gene expression between cultivars ‘Carene’ and ‘Big Bang’ before storage, and between ‘Carene’ and ‘Big Top’ after cold storage.

Of specific interest were genes related to the major VOC families identified from peach aroma: fatty acid-derived esters, ketones, terpenes, straight-chain alcohols, lactones, and aldehydes.

**Figure 4:**
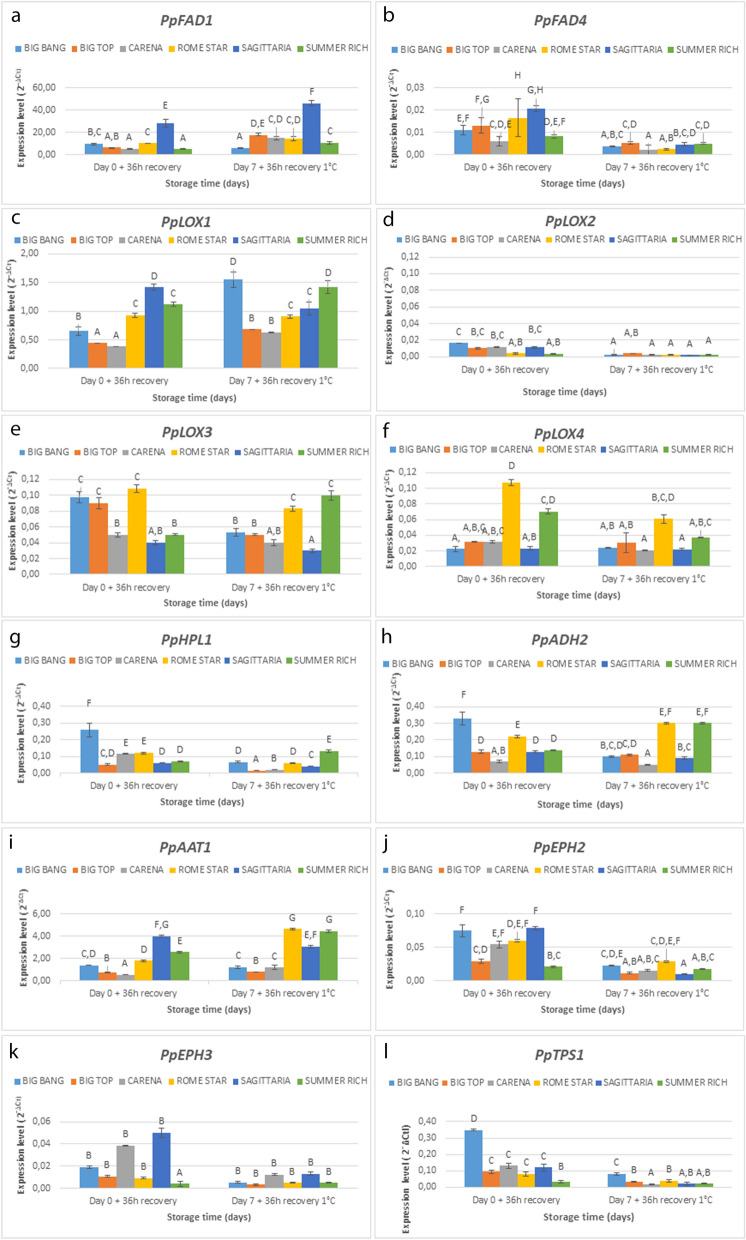
Real-time PCR analysis of selected differentially expressed genes related to VOC compounds, among six peach/nectarine cultivars analysed at two time points day 0 (before) and day 7 (after) cold storage (**a**) *PpFAD1*: Fatty acid desaturase 1 (FAD); (**b**) *PpFAD4*: Fatty acid desaturase 4 (FAD); (**c**) *PpLOX1*: lipoxygenase 1 (LOX); (**d**) *PpLOX2*: lipoxygenase 2 (LOX) (**e**) *PpLOX3*: lipoxygenase 3 (LOX); (**f**) *PpLOX4*: lipoxygenase 4 (LOX); (**g**) *PpHPL1*: hydroperoxide lyase (HPL), (**h**) *PpADH2*: alcohol dehydrogenase 2 (ADH) (**i**) *PpAAT1*: alcohol acyl transferase (AAT); (**j**) *PpEPH2*: Epoxide hydrolase enzymes (EPH); (**k**) *PpEPH3* Epoxide hydrolase enzymes (EPH); (**l**) *PpTPS*1: terpene synthase 1 (TPS); *PpTEF2* was used as an internal control to normalize small differences in template amounts according to Tong et al.^64^. Letters indicate significant differences among cultivars considering all time points. Statistical analyses were performed using ANOVA and Tukey’s ranked test (*P* < 0.05). Data are the mean ± SE; n = 3.

Relating to the production of lactone and ester VOCs, of the two *FAD* genes, analysed (Fig. 4a,b), *PpFAD1* was much more highly expressed than *PpFAD4* in all the cultivars. In all cultivars apart from ‘Big Bang’ expression of *PpFAD1* increased significantly following cold storage, with the highest expression both before and after cold storage seen in ‘Sagittaria’. Differences in expression amongst the cultivars were also more evident after cold storage. The opposite trend was observed for *PpFAD4* whose expression level decreased following cold storage in all the cultivars. Also for this gene, there were clear differences amongst cultivars both before and after cold storage, although differences were more marked before cold storage.

In relation to the LOX pathway, expression analysis of four *LOX* genes (*PpLOX1*, *PpLOX2*, *PpLOX3*, and *PpLOX4*) revealed differences in expression amongst the genes, cultivars and in response to the storage treatment (Fig. 4c–f). *PpLOX1* expression increased following cold treatment in all three nectarine cultivars, whereas in the peach cultivars under the same storage conditions gene expression was more variable. Differences in the expression of this gene were evident both with and without cold storage: there was greater expression in the peach cultivars without cold storage, but after cold storage expression was highest in ‘Big Bang’ and ‘Summer Rich’. All three of the other LOX genes analysed (*PpLOX2*, *PpLOX3* and *PpLOX4*), generally showed reduced expression in response to cold storage although the differences were not significant for all the cultivars. The only exception was the twofold relative increase in expression of *PpLOX3* transcripts in ‘Summer Rich’ peaches. Following cold storage, expression of *PpHPL1* decreased significantly in all cultivars except ‘Summer Rich’ where again the opposite trend was found (Fig. 4g). Again, there were differences in expression across the cultivars both with and without cold treatment.

Concerning the production of alcohol VOCs, overall, *PpADH2* gene expression differed in relation to the cultivar more than in relation to treatment (Fig. 4h). Significant cultivar-dependent changes in *PpADH2* expression were induced following cold-storage. The level of *PpADH2* transcript decreased in early cv.s ‘Sagittaria’ and ‘Big Bang’, it increased in cv.s ‘Rome Star’ and ‘Summer Rich’, although the increase was only significant in the latter (Fig. 4h). No significant variation was found for cv.s ‘Big Top’ and ‘Carene’.

*PpAAT1* transcript levels, related to the production of ester VOCs were significantly upregulated following cold storage in nectarine cv. ‘Carene’ and the late ripening *cv.s* ‘Rome Star’ and ‘Summer Rich’ peaches (Fig. 4i).

Expression of *PpEPH2* and *PpEPH3,* encoding enzymes involved in lactone production was down regulated by cold storage in all the cultivars, although the differences were not always statistically significant (Fig. 4j,k).

Finally, expression of *PpTPS1* involved in terpene biosynthesis fell significantly in almost all cultivars in response to cold storage (Fig. 4l). *PpTPS2* transcripts were not detected in any of samples analysed.

### Correlation between changes in VOC abundance and gene expression in the LOX related pathway

Weighted Correlation Network Analysis (WCNA) was used to investigate correlations between changes in abundance of 63 of the 115 VOCs, which are related to the LOX pathway including alcohols, ketones, aldehydes, esters and lactones, and gene expression of 11 enzymes of the LOX pathway. These were: fatty acid dehydrogenases (*PpFAD1*, *PpFAD4*), lipoxygenases (*PpLOX1-4*), epoxide hydrolases (*PpEPH2*, *PpEPH3*), hydroperoxide lyase (*PpHPL1*), alcohol dehydrogenase (*PpADH2*) and alcohol acyl transferase (*PpAAT1*).

**Figure 5:**
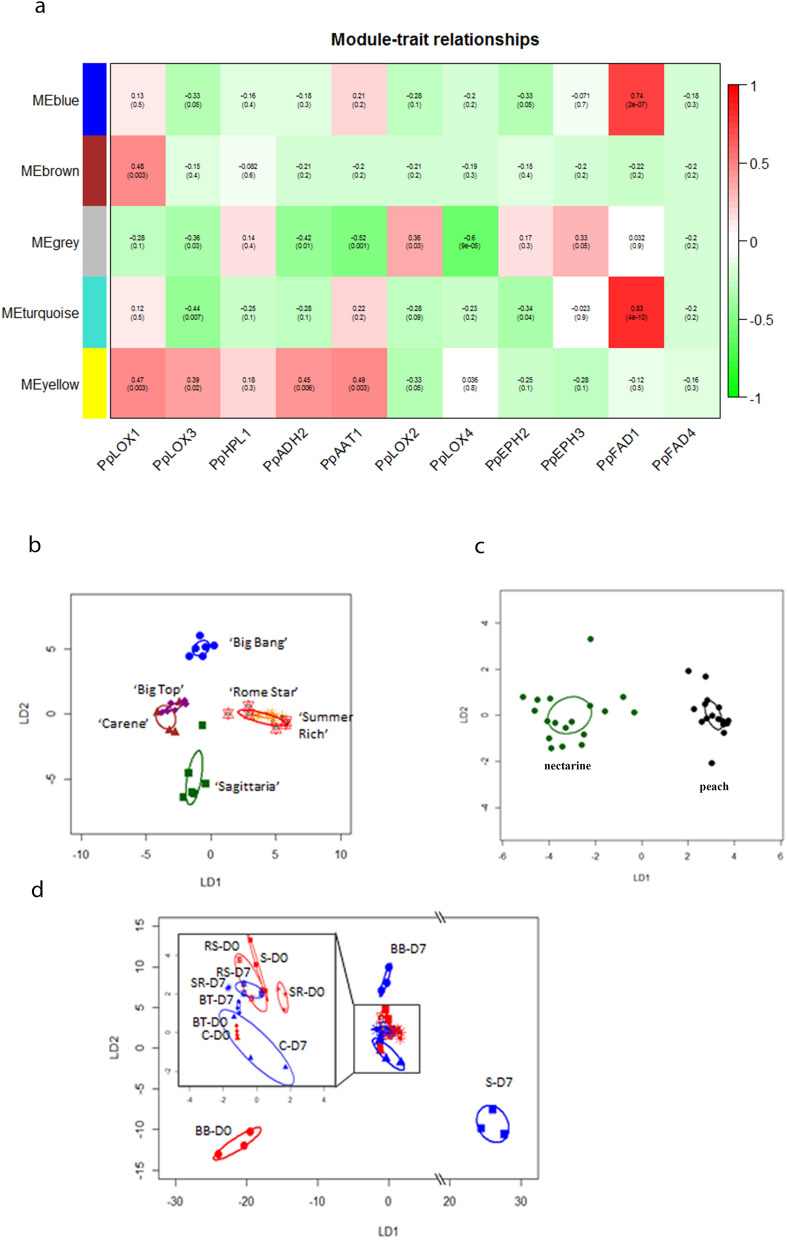
Multi-trait analysis of expression genes from LOX pathway and 63 associated LOX pathway VOCs in the six peach cultivars before and after cold storage (**a**) Heat map of WCNA modules with scores and significance (*P* values in brackets) based on a Pearson analysis; (**b**–**d**) linear discriminant plots from Canonical Analysis of Principal coordinates (CAP) based on 16 VOCs selected from WCNA analysis of LOX gene expression versus LOX related VOCs from six peach cultivars: ‘Sagittaria’, ‘Big Bang’, ‘Big Top’, ‘Carene’ ‘Rome star’ and ‘Summer Rich’, by (**b**) cultivar, (**c**) peach versus nectarine, (**d**) the six peach cultivars analysed before (D0, in red) and after (D7, in blue) a 7 day storage period at 1 ℃ followed by a 36 h shelf life storage at 20 ℃. (mean + SE). Each ellipse represents the 95% confidence interval of SE. The plots use LD1 (**b**) with a percentage of correct classification = 83% (*P* = 0.01; n = 6); (**c**) 97% (*P* = 0.01; n = 18); (**d**) 72% (*P* = 0.9; n = 3).

WCNA clustered the 63 VOCs into five modules (Fig. 5a: blue: seven VOCs, brown: five VOCs, grey: 32 VOCs, turquoise: 15 VOCs and yellow: four VOCs). Different VOC families were present in the five modules and there was no discernible pattern of the families amongst modules. Four compounds did not correlate significantly with any gene expression (Supplementary Table S2). Overall, 39 of 55 correlations were negative and only 13 correlations were statistically significant (Fig. 5a).

The strongest correlations were found between *PpFAD1* and blue and turquoise modules (positive), and *PpLOX4* and the grey module (negative) while no significant correlations were found for *PpHPL1* and *PpFAD4*. *PpLOX1* was correlated positively (0.01 > *P* > 0.001) with the yellow and brown module, while *PpLOX2* was positively correlated to the grey module (Fig. 5a). The turquoise module was affected by expression of *PpLOX2* (positive), *PpLOX3* (negative) and *PpEPH2* (negative) and the grey module by expression of *PpLOX3*, *PpADH2*, *PpAAT1, PpLOX2* and *PpLOX*4 where all genes except *PpLOX2* were negatively correlated (Fig. 5a). The brown and blue modules only correlated strongly and significantly with expression of a single gene: *PpLOX1* (brown, positive) and *PpFAD1* (blue, positive) (Fig. 5a). Only two genes (*PpFAD1* and *PpLOX4*) showed strong correlations between expression and abundance of VOCs, and an increased expression of *PpFAD1* correlated positively to all compounds of the blue and 14 out of 15 of the turquoise module (Supplementary Table S3). All six compounds in the blue module showed a highly significant positive correlation with *PpFAD1* as did 12 of the 15 compounds in the turquoise module (Supplementary Table S3).

Sixteen VOCs were selected from the WCNA modules based on their high correlation with the LOX gene expression associated with each module, with numbers of VOCs selected from each module proportionate to the total number of VOCs in that module (Table 3; Supplementary Table S3). When these VOCs were used for PerMANOVA they showed a significant correlation with cultivar (R^2^ = 0.17, *P* < 0.001), and day (R^2^ = 0.07, *P* < 0.01). Linear discriminant analysis using these 16 VOCs separated the majority of cultivars apart from ‘Carene’ from ‘Big Top’ and ‘Summer Rich” from “Rome Star” when both time points were considered together (Fig. 5b). They also discriminated peach versus nectarine, both combining the two time points (Fig. 5c) and considering separately before and after storage (Supplementary Fig S2e, f online). This sub-set of VOCs also separated most individual cultivars before and after cold storage apart from ‘Carene’ and ‘Rome Star’ (Fig. 5d).

**Table 3.** 16 VOCs selected from the LOX pathway based on their correlation with LOX gene expression in WCNA analysis.

VOC code	VOC name	Family
C35	*cis*-3-hexenyl acetate	Acetate ester
C132	Butyl carbitol acetate	Acetate ester
C41	(E)-2-Hexenyl acetate	Acetate ester
C138	2-methyl-1-decanol	Alcohol
C96	1-*p*-Tolylethanone	Ketone
C139	Tetrahydrogeranylacetone	Ketone
C144	Geranyl acetone	Ketone
C128	6-Dodecanone	Ketone
C60	γ-Caprolactone	Lactone
C150	γ-Decalactone	Lactone
C120	Amyl caproate	Non-Acetate ester
C172	2-Ethylhexyl 4-methylbenzoate	Non-Acetate ester
C161	Pentanoic acid‚ 2‚2‚4-trimethyl-3-carboxyisopropyl‚ isobutyl ester	Non-acetate ester
C74	Methyl 4-octenoate	Non-acetate ester
C106	*cis*-3-Hexenyl valerate	Non-acetate ester
C76	Methyl octanoate	Non-acetate ester

### Correlation between terpene VOCs and expression of the terpene synthase PpTPS1

Based on their change in abundance over time across the six cultivars, the fourteen terpenes that were detected in the peach cultivar volatilomes were correlated with the expression of *PpTPS1* using WCNA. Seven compounds clustered together and were positively correlated with gene expression (Pearson corr. 0.89, *P* < 0.0001). Four compounds, linalool (C69), cosmene (C78), caryophellene (C145) and humulene (C146), were highly significantly and positively correlated with expression of *PpTPS1* (Supplementary Table S4).

## Discussion

Relatively few studies have examined changes in aroma profiles of intact peach fruit in relation to cold storage^25,48,49^. Brizzolara and Tonutti^49^ reported only 25 compounds across three peach cultivars employing SPME-GC-MS, and 28 were reported using diethyl ether extraction of VOCs from whole cv. ‘Tardibelle’ peaches followed by GC-MS^25^. On the other hand, using TD-GC-MS^48^, identified 115 VOCs from cv. ‘Springcrest’. Surprisingly, although a similar number of VOCs were identified in this study, only 18 were common to those identified by Malorni et al.^48^, and only seven were in common between our study and that of Brizzolara and Tonutti^49^. These differences in the number of VOCs and VOC composition between our study and previous studies may be due to a number of factors. These include the different varieties used, the GCxGC separation approach used here, or alternatively might be ascribed to seasonal variation, since this has been found to affect aroma composition and detection of individual VOCs^10^.

Differences were also found in the VOC family composition between our study and previous studies. Although almost all the same major VOC families are represented in our study and in Malorni et al*.*^48^, the major differences were in the greater number of acids found in Malorni et al.^48^, and ketones and terpenes in our aroma profiles. In contrast, the aroma profiles reported for Brizzolara and Tonutti^49^ for cv.s ‘Flaminia’, ‘Regina di Londa’, and ‘Red Haven’ were dominated by acetate esters, alcohols, ketones and lactones, while those reported in Ortiz et al.^25^ are dominated by non-acetate esters with high proportions also of acetate esters and alcohols. These differences amongst studies may be due to the cultivars tested, or the contrasting analysis methods.

An important component of peach aroma are the key odour active volatile (OAV) compounds. All three VOCs common to this and other whole peach aroma studies^25,48,49^, (hexyl acetate, γ decalactone and linalool) were identified as a key peach OAVs^9^ and have low odour thresholds (of 0.002, 0.011 and 0.006 mg/L; Bononi et al.^50^. However, of the 30 VOCs identified in Zhu and Xiao^9^ from peach musts with OAVs of > 1 only eight were identified here and a further eight in at least one of the three other aroma analyses from whole peaches^25,48,49^. Of the ten VOCs identified by Zhu and Xiao (2019)^9^ as those with the most powerful aroma, only four were represented in profiles here (linalool (C69), (E)-2-octenal (C71), (E)-2-nonenal (C100) and γ decalactone (C150)) and one further VOC (hexanal) was represented in three whole peach studies^25,48,49^. The five cv.s analysed by Zhu and Xiao^9^ were all Chinese, which might account for this difference to the whole peach studies. However, nine other cv.s analysed by Eduardo et al*.*^6^ for their OAV analysis included cv. ‘Big Top’, one of the cv.s analysed here, and again few VOCs were in common. This lack of the reported OAVs characteristic of peach aromas in studies using whole peaches may be due to cultivar differences, however, an important factor is that the 21 OAVs in Eduardo et al*.*^6^ were identified from peach essential oil extracted by steam distillation followed by solvent separation. Furthermore Zhu and Xiao^9^ analysed refrigerated peach musts using SPME. Both of these approaches are likely to result in substantial modifications to the VOC profile composition compared to fresh peaches. Hence the OAV content in the aroma from whole peaches may be very different from that seen when peaches are not intact.

The whole VOC profiles presented here were able to clearly discriminate the six cultivars tested, and between nectarines and peaches before storage, in agreement with other studies^4^. The peaches had significantly more lactones in their VOC profile suggesting they might also be classed in the first group identified in Wang et al*.*^10^ as producing aromas with high lactone content, although acetate esters and ketones were also high in the peaches here. However, after storage the discrimination between nectarines and peaches using the whole VOC profile was lost, indicating that changes induced during the storage period result in more similar VOC profiles. Nevertheless, it was possible to identify a subset of 15 VOCs by Random Forest that retained discrimination even after cold storage, and the subset of 16 LOX-related VOCs also clearly discriminated nectarines from peaches both before and after cold storage. The 16 LOX-related VOCs included two VOCs also found amongst the top 15 VOCs identified by Random Forest as being the most discriminatory between nectarines and peaches, specifically γ-caprolactone (C60) and γ-decalactone (C150). The latter has a high OAV^9^ indicating that it might be important in sensory discrimination between these varieties.

The VOC pattern when all cultivars were grouped together was not distinct between fruit before and after storage. This appears to contrast with earlier work^24^ showing that stored fruit had a distinctive VOC profile. However, the experiments differed importantly in the temperature of storage and storage time, with distinctive profiles developing most clearly only after a longer period of storage, and at 5 °C, a temperature, which promotes chilling injury. Moreover, Zhang et al*.*^24^ only studied a single peach cv., and here the VOC profiles of most individual cv.s was distinct before and after storage. Therefore, assessing changes in VOCs across multiple cv.s in relation to storage is important since both initial profiles, and the changes elicited by cold storage varies with cv.

The abundance of some VOC classes changed significantly across all cv.s examined here after storage. The increase in lactones with short term storage (up to a week) at low temperature (0–2 °C) is in agreement with previous studies using other cultivars^19,24,51^. In peach, *PpLOX1* and *PpLOX4* were found associated with the synthesis of lactones^33^. This is consistent with the positive correlation of *PpLOX4* expression with γ-caprolactone (C60) and γ-Decalactone (C150) that we detected using WCNA. The WCNA analysis also correlated high *pPLOX4* expression with a high level of γ-decalactone (C150), specifically in late ripening ‘Rome Star’ and Summer Rich’. These results suggest that these lactones, and mainly γ-decalactone, may be regulated by transcriptional changes in *pPLOX4,* at least in these late ripening cultivars. No correlation with LOX gene expression was detected for the other three lactones identified in the VOC analysis (δ-nonalactone (C156), butyrolactone (C63) and γ-nonalactone (C136)) indicating that their abundance may depend on other genes involved in the biosynthetic pathway.

The expression of the LOX genes across the six cv.s in response to cold differed. In agreement with Zhang et al.^24^ *PpLOX1* increased in expression following 7 days of cold storage plus recovery period of shelf life storage at 20 ºC in the fruit of the three nectarine cultivars. However, the expression in the peaches was different, indicating perhaps a different role for this gene in these two types of fruit. Changes in *PpLOX4* expression with storage here contrasted with the rise seen following cold storage by Zhang et al*.*^24^, again perhaps reflecting cv. specific roles of the different LOX genes. In contrast, the fall in *PpLOX2* across all cv.s here is in agreement with previous findings^24^ and perhaps indicates a cultivar-independent role for this gene.

In peach PpFAD1 is also linked to the biosynthesis of lactones^30,39^. Here, *PpFAD1* was much more highly expressed than *PpFAD4* irrespective of storage condition. The positive correlation of *PpFAD1* expression with γ-nonalactone (C136) based on WCNA indicates that this enzyme may also contribute to modulation of lactones in the VOC profile after cold storage. Moreover, while *PpFAD1* was positively correlated (*P* < 0.001), with its WCNA module, *PpLOX3* and *PpEPH2* were negatively correlated (*P* < 0.001 and *P* < 0.05 respectively). This result appears to be in line with the negative interaction between the lactones and lipid-derived VOCs previously reported^46^.

Aldehydes and non-acetate esters also increased following cold storage across the six cv.s tested here, and the positive correlation of *PpFAD1* expression with several non-acetate and acetate esters, based on WCNA, suggests the possibility that transcriptional regulation of this enzyme may be important in VOC profile modulation across different VOC families. Expression of other genes from the LOX pathway, related to the production of VOC alcohols and aldehydes were also affected by the cold storage. In line with Zhang et al*.*^24^, following cold storage, expression of *PpHPL1* decreased significantly in all cultivars with the only exception of ‘Summer Rich’ peach, for which gene expression exhibited an opposite trend. Again consistent with Zhang et al*.*^24^, *PpADH2* gene expression, also declined following cold storage in all cv.s, except for ‘Rome Star’ and ‘Summer Rich’. This is also consistent with a decline in ADH activity in the fruit of ‘Rich Lady’ peach cultivar during shelf-life^52^. In tomato, over-expression of Le-ADH2, increased the level of alcohols, particularly Z-3-hexenol^53^. Here the fall in *PpADH2* expression did not correlate with a loss of alcohols although in some cultivars there was a slight but not significant reduction in overall alcohol VOC relative abundance. No C6 alcohols were detected in the VOC profiles here, although hexanols were detected in other studies that analysed whole peaches including cv. ‘Big Top’^14^ where the relative abundance of 1-hexanol did fall with storage at − 0.5 °C for 10 days followed by 3 days shelf life storage at 20 °C. Differences in the detection of C6 alcohols may be seasonal or due to the detection method used.

In contrast to *PpHPL1* and *PpADH2,* transcript levels of *PpAAT1,* also involved in the LOX pathway*,* were significantly upregulated following cold storage in nectarine cv. ‘Carene’ and in peaches cv.s ‘Rome Star’ and ‘Summer Rich’. This is in line with data previously reported for cv. Hujingmilu stored for 7 days at 0 °C with 3 days of shelf life storage at 20 °C, where *PpAAT1* transcript levels increased 3-fold^24^. A strong positive correlation was found in the WCNA between *PpAAT1* and (Z)-3-hexenyl acetate (C35) but not with hexyl acetate (C39) or (E)-2-hexenyl acetate (C41) that all derive from the lipoxygenase pathway^30^. This suggests that expression of this gene may be important in the biosynthesis of (Z)-3-hexenyl acetate.

Regarding terpene VOCs, a close significant intra-module positive correlation was found here between *PpTPS1* and linalool (C69) by WCNA. After cold treatment *PpTPS1* transcript level decreased, correlating with a reduction in linalool during storage as was also previously noted^45,51^. Although the change in linalool with storage here was not statistically significant in any of the cv.s, due to variability, the correlation between loss of linalool and reduction in *PpTPS1* expression supports the likely role of PpTPS1 in the biosynthesis of linalool^44^.

Thus overall, VOC profile, gene expression and changes in both characters with cold storage appear to be cultivar specific. Indeed, we show here that potential markers for cold storage that can be derived from a subset of the total VOC profile are also likely to be cultivar specific. This indicates the need to test both VOC and gene markers across a wide range of cultivars to assess robustness for commercial detection of chilling conditions during transport. Since responses to cold storage are cultivar specific, this indicates the opportunity for breeding and selection targeted at retention of VOC profile after storage. The correlation between gene expression and VOC profiles in different cultivars is useful in revealing potential biosynthetic relationships. However, the gene response and VOC profile changes seen here were not always in complete agreement: for example the VOC profile of cv. ‘Big Bang’ was not significantly affected by the storage regime imposed, whereas the expression of the genes tested was. This suggests that other factors such as enzyme activity during storage may provide resilience to VOC changes despite changes in transcription.

## Materials and methods

### Fruit material and post-harvest conditions

The following melting and yellow peach cultivars (*Prunus persica* (L.) Batsch) and nectarine (*P. persica* var. *nucipersica*), were selected to include different ripening periods in May/early June (early ripening), June (mid season ripening) and late July (late season ripening). ‘Sagittaria’ is an early ripening melting, yellow flesh, clingstone peach, while ‘Big Bang’ is an early ripening melting, yellow flesh clingstone, nectarine, ‘Big Top’ and ‘Carene’ are middle ripening yellow flesh, nectarines with ‘Big Top’ being melting flesh and semi-free stone, and ‘Carene’ stony-hard and freestone, while ‘Rome Star’ and ‘Summer Rich’ are late ripening, melting, yellow flesh, freestone peaches. All cultivars were grown at the "Campo Verde" Agricultural Company, Calabria, Italy [(39° 48′ 58″ N, 16° 12′ 06″ E, 382 m above sea level, (ASL)]. Sampling was carried out in the 2017 summer season. Commercially mature fruits were collected manually and for each cultivar 20 kg of fruit, (about 100 fruits), were selected for uniformity in size, maturity, appearance (colour and size) and lack of defects and then transported to the laboratory.

To verify the assessment of maturity stage fruits, considered of equal maturity based on appearance, were tested for flesh firmness (kg cm^−2^) and total soluble solids (°Brix) (Supplementary Table S5). Total soluble solids were measured using an optical refractometer MA871 (Milwaukee, Rocky Mount, NC, USA). Flesh firmness was assessed on two sides of fruits sampled, after the removal of a 1 mm thick slice of skin, using an FT30 (Wagner, Greenwich, CT, USA). Values were within the range previously described for commercially mature fruit^54^.

After harvesting, for each cultivar one batch (D0) was immediately transferred to 22 °C for 36 h to reach the fully ripe stage (boxes of 30 fruits). A second batch of fruit (D7) was stored for 7 days at 1 °C and then transferred to 22 °C for 36 h to allow the development of the delayed ripening phase. Assuming a typical supply chain where peach fruits are imported by lorry from Southern Italy to Northern Europe, 7 days were selected as the mean time taken from harvest to retail, while 1 °C was chosen as a commercially relevant storage temperature^55^.

For each cultivar and for each post-harvest storage condition (D0 and D7) sub-samples were immediately processed for VOC analysis as detailed below. The remaining fruit were peeled, and slices of mesocarp (about 1 cm thick) were excised, pooled, frozen in liquid nitrogen, and stored at − 80 °C for molecular analyses. All the analyses were carried out on triplicate samples (5 fruits for each replicate).

### Sample collection and analysis of VOCs

Whole peaches were placed in a Nalophene bag and left to equilibrate at 20 °C for 90 min prior to sampling. Headspace (600 mL) from within the roasting bag was then drawn directly onto an ‘Odour/Sulfur’ sorbent tube (Markes International, Llantrisant, UK) using an Easy-VOC manual pump (Markes International, Llantrisant, UK). The process was also performed on empty roasting bags as control blanks. Thermal desorption (TD) was carried out using a TD100-xr (Markes International, Llantrisant, UK) with a tube desorption temperature of 280 °C for 10 min and trap collection at 25 °C and desorption of trap at 300 °C for 5 min. An outlet split ratio of 41:1 was used. GC × GC was performed using an INSIGHT flow modulator (SepSolve Analytical, Peterborough, UK) mounted within a 7890B GC (Agilent Technologies, Santa Clara, CA, USA). A loop volume of 25 μL was used with a fill time of 3920 ms and flush time of 80 ms to give a total modulation period (P_M_) of 4.0 s. The column set consisted of a BPX5 (20 m × 0.18 mm × 0.18 μm) and MEGA WAX-HT (5 m × 0.32 mm × 0.1 μm). The GC oven was programmed at 40 °C (hold 3 min), 5 °C/min to 280 °C (hold 10 min).

Detection was performed using a BenchToF-Select time-of-flight mass spectrometer (Markes International, Llantrisant, UK). The ion source temperature was set to 350 °C and transfer line to 300 °C. A mass range of m/z 45–350 was used for all measurements.

### Analysis of GCxGC-ToF-MS data

GC × GC data processing was carried out using ChromSpace (SepSolve Analytical, Peterborough, UK). Peaks were identified by screening against the NIST17 spectral library (NIST v. 2.3, 2017). VOC data were normalised to the grand-total of peak areas and square root transformation was applied to reduce weight of large components. Non detected components (missing values) were replaced with 1/10 of the minimum value across all samples. Statistical analysis was then performed using PerMANOVA and Canonical Analysis of Principal coordinates (CAP)^56^ using the ‘vegan’ and ‘BiodiversityR’ packages v. 1.2 in R software v. 3.6.2^57^ and MetaboAnalyst 3.03^58^. Weighted correlation network analysis (WCNA) was used to correlate gene expression with VOC profile with a soft-power setting of 10^58^. Random Forest (RF) is a supervised machine-learning algorithm used in data-rich fields such as bioinformatics or chemo-informatics to select the most appropriate classification variables from surrounding noise in a highly variable dataset^60,61^. It uses an ensemble of classification trees, each of which is grown by random feature selection from a bootstrap sample at each branch. RF also provides other useful information such as a variable importance measure, and outlier measures. During tree construction, about one-third of the instances are left out of the bootstrap sample. Three classifications were performed using the RF within Metaboanalyst to differentiate across all cultivars, cold treatment, and peaches from nectarines. The RF algorithm was used to find the smallest set of predictor VOCs for each classification.

All the compounds were identified by comparison to NIST libraries. Comparison of VOC family relative abundance was analysed using ANOVA followed by a Tukey’s test, or if the data did not satisfy the normality and homoscedasticity assumptions, Kruskall-Wallis multiple comparison test and post hoc Dunn's test were applied instead.

### RNA extraction

Peach mesocarp slices from five fruits for each biological replicate were powdered with liquid nitrogen using a food processor (Bimby, Vorwerk, Germany). About 100 mg of the peach mesocarp powder were used for total RNA extraction using an Agilent Total RNA Isolation Mini Kit (Agilent Technologies, Santa Clara, CA, USA) according to the manufacturer’s instructions. Each RNA sample was treated with RNAse-free DNAse (Qiagen, Hilden, Germany). The purity and concentration of the extracted RNA was checked using a NanoDrop (ND-1000 UV–Vis spectrophotometer; NanoDrop Technologies, Wilmington, DE, USA), while its integrity was checked on an Agilent 2100 Bioanalyzer RNA Nano Chip (Agilent Technologies, Santa Clara, CA, USA). Only RNA samples with an RNA integrity number ≥ 8 were used for real time Q-PCR.

### Real time PCR analysis

RNA (100 ng) from each sample were retro-transcribed into cDNA using the iScript cDNA synthesis kit (Invitrogen, Monza, Italy), according to the manufacturer’s protocol. Gene expression analyses were carried out on a STEP ONE instrument (Applied Biosystems, Monza, Italy) by using Power SYBR Green PCR Master Mix 2X (Applied Biosystem, Monza, Italy). Amplification reactions were prepared in a final volume of 20 µl by adding 2.5 µl Power SYBR Green PCR Master Mix, 2 µl of cDNA (40 ng) and 1 µl each primer (0.2 µM). All reactions were run in triplicate in 48-well reaction plates and negative controls were included. Melting curve analysis was also performed. The cycling parameters were as described in Bruno et al*.*^62^.

The primer sets used are listed in Supplementary Table S6 online: twelve genes related to VOC biosynthetic pathways were selected from the literature^33,44,63^ and *PpTEF2* was used as an internal control to normalize small differences in template amounts according to Tong et al.^64^. Relative quantification of gene expression was calculated according to Schmittgen and Livak^65^. First, ΔCt values were obtained by calculating, for each cultivar, the difference of the Ct of each target compared to the arithmetic mean of the Ct for the housekeeping gene (*PpTEF2*). Then, the relative expression was expressed as (2^−ΔCt^). Statistical analyses were performed using ANOVA and a Tukey’s rank test (*P* < 0.05) on ΔCt values and lower case letters on graphs indicate significant differences.

## Supplementary information

Supplementary Information 1.

Supplementary Information 2.

Supplementary Information 3.

Supplementary Information 4.

Supplementary Information 5.

Supplementary Information 6.

Supplementary Information 7.

Supplementary Information 8.

Supplementary Information 9.

## Data Availability

All key data are provided in the Supplementary Files. Further primary datasets generated during and/or analysed during the current study are available from the corresponding author on reasonable request.
