# Supplementary material for: Fruit volatilome profiling through GC × GC-ToF-MS and gene expression analyses reveal differences amongst peach cultivars in their response to cold storage
Source: Sci Rep. 2020 Oct 27;10:18333. doi: 10.1038/s41598-020-75322-z (PMC7591569; doi:10.1038/s41598-020-75322-z)
Supplement: Supplementary file 1 — Supplementary Information 1. [file 41598_2020_75322_MOESM1_ESM.docx]

**SUPPLEMENTARY TABLES:**

**Supplementary Table 1a,b:** (a) complete list of VOCs detected in all peach samples; (b) relative abundance of each VOC detected across the 6 peach cv.s.

**Supplementary Table S2:** Output from Weighted Correlation Network Analysis of VOCs and gene expression related to the LOX pathway.

**Supplementary Table S3:** Summary of correlations between gene expression and modules derived from WCNA of gene expression and VOCs from the LOX pathway.

**Supplementary Table S4:** Output from WCNA analysis of gene expression and VOCs from the terpene pathway.

**Supplementary Table S5:** Acidity and Brix in commercially ripe peaches

**Supplementary Table S6:** Primers used in the present study.

**SUPPLEMENTARY FIGURE LEGENDS:**

**Supplementary Fig. 1:** GCxGC-TOF MS colour plots showing separation of peaks that would have coeluted in a 1D GC separation (13.88 min). Rome Star, Day 7, *cis*-3-Hexenyl acetate (C33), α-Phellandrene (C34).

**Supplementary Figure S2:** Linear discriminant plots from Canonical Analysis of Principal coordinates (CAP) based on all VOCs analysed using TD-GCxGC-TOF-MS from six peach cultivars combining before (day 0) and after (7 day). (a) discrimination by day of storage (b) discrimination between nectarine and peach cultivars; (c) and (d) use only the top 15 discriminatory VOCs from Random Forest analysis (shown in Fig. 2a) by (c) peach vs nectarine before and (d) after cold storage; (e) and (f) use 16 VOCs selected from WCNA analysis: peach vs nectarine (e) before (Day 0) and (f) after (Day 7) cold. Each ellipse represents the 95 % confidence interval. The plots use LD1with a percentage of correct classification of: (a) 75 %, (n=18, ±SD); (b) 91.6 % (P=0.01, n=18, ± SD); (c) 94% (P=0.01, n=9, ± SD); (d) 89% (P=0.02, n=9, ± SD); (e) 89% (P=0.01, n=9, ± SE); (f) 94.4% (P=0.01, n=9, ± SE).
