# Supplementary material for: Fruit volatilome profiling through GC × GC-ToF-MS and gene expression analyses reveal differences amongst peach cultivars in their response to cold storage
Source: Sci Rep. 2020 Oct 27;10:18333. doi: 10.1038/s41598-020-75322-z (PMC7591569; doi:10.1038/s41598-020-75322-z)
Supplement: Supplementary file 7 — Supplementary Information 7. [file 41598_2020_75322_MOESM7_ESM.pdf]

Supplementary Table S6: Primers used in the present study.

PATHWAY	Gene	annotation	GenBank accession number/Peach EST database accession number	References	forward primer (5'-3')	reverse primer (5'-3')
Six carbon aldehydes and alcohols	*PpLOX1*	Lipoxygenase	EU883638	*Zhang . et al., 2010*[33]	GGCAATTAGGAAAAGTGGTTG	TGCGCAATATTTTTGGTGTG
Six carbon aldehydes and alcohols	*PpLOX2*	Lipoxygenase	FJ029110	*Zhang . et al., 2010*[33]	GTGGACTCACTGGGAGAGGA	GTTGCACGACCATTTCACAC
Six carbon aldehydes and alcohols	*PpLOX3*	Lipoxygenase	FJ032015	*Zhang . et al., 2010*[33]	CATCCTTCTAGTGAGGCTGGA	GGAGAAGCATTAAATTGAGACACTG
Six carbon aldehydes and alcohols	*PpLOX4*	Lipoxygenase	EF568783	*Zhang . et al., 2010*[33]	TCCTTCTCTCTTGGCTCGAT	GGGCTTGCATTCGCATAGTA
Aldehydes	*PpHPL1*	hydroperoxide lyase	DW354957/	*Zhang . et al., 2010*[33]	AAATGGGGATGTGATGGATG	TTGCCCTTTTCCCTCAAGTA
Alcohols	*PpADH2*	alcohol dehydrogenase	DY641017	*Zhang . et al., 2010*[33]	GATTTGATGCTTTCAGGACAGT	CCAAGTTGCTCTAATCCTCCA
Ester	*PpAAT1*	Alcohol acyl transferase	DY645545	*Zhang . et al., 2010*[33]	TTGGAGAGGTTTGAGGAGGA	AGCCCACACAACACAAGACA
Polyunsaturated fatty acid	*PpFAD1*	Fat acid desaturase	AJ824111	*Zhang . et al., 2010*[33]	CGGTTTTCAAGGCAATGTTC	CCTACACTCATTCGGGCAAT
polyunsaturated fatty acid	*PpFAD4*	Fat acid desaturase	AJ876286	*Zhang . et al., 2010*[33]	ACGTTGCCTTTGACCAACTT	AATGACTGTGACCCCACCAC
Lactones	*EPH2 lattoni*	Epoxide hydrolase-like protein	ppa008854m	*Pirona et al., 2012*[63]	TTTCAGGTCTGGCACAAGACTCT	TGATTGTAGGTGACAAAGACATTGG
Lactones	*EPH3 lattoni*	Epoxide hydrolase-like protein	ppa009153m	*Pirona et al., 2012*[63]	TCACCCACAAGGCTCTGCTT	ACTTCTCGCCTATCTTCCTATATATCTGA
Terpens	*PpTPS1*	Terpene synthases	Prupe.4G030400	*Liu et al., 2017*[44]	TCAACGGCTGGGTATTGACC	TGAGCAGTCGAAAGCGAACT
Terpens	*PpTPS2*	Terpene synthases	Prupe.4G029900	*Liu et al., 2017*[44]	GCTCAGCAATGATTTGGGAACTTCT	TGTGGTAATGTTGATGAATGGTGA
Housekeeping	*PpTEF2*	Translation elongation factor 2H	TC3544	*Tong et al., 2009*[64]	GGTGTGACGATGAAGAGTGATG	TGAAGGAGAGGGAAGGTGAAAG
