# Supplementary figures and images for: Fruit volatilome profiling through GC × GC-ToF-MS and gene expression analyses reveal differences amongst peach cultivars in their response to cold storage
Source: Sci Rep. 2020 Oct 27;10:18333. doi: 10.1038/s41598-020-75322-z (PMC7591569; doi:10.1038/s41598-020-75322-z)

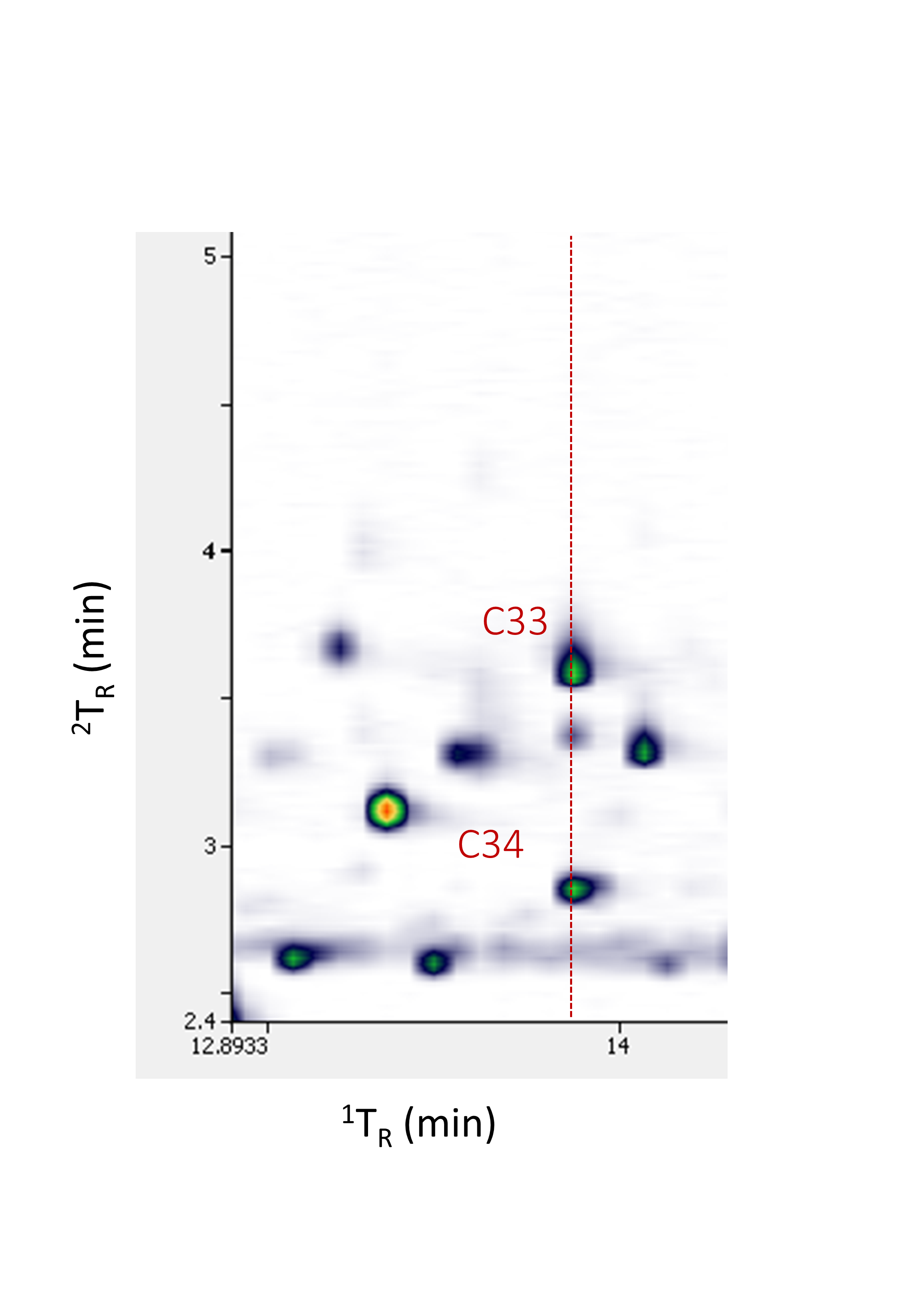

Supplement: Supplementary file 8 — Supplementary Information 8. [file 41598_2020_75322_MOESM8_ESM.tif]

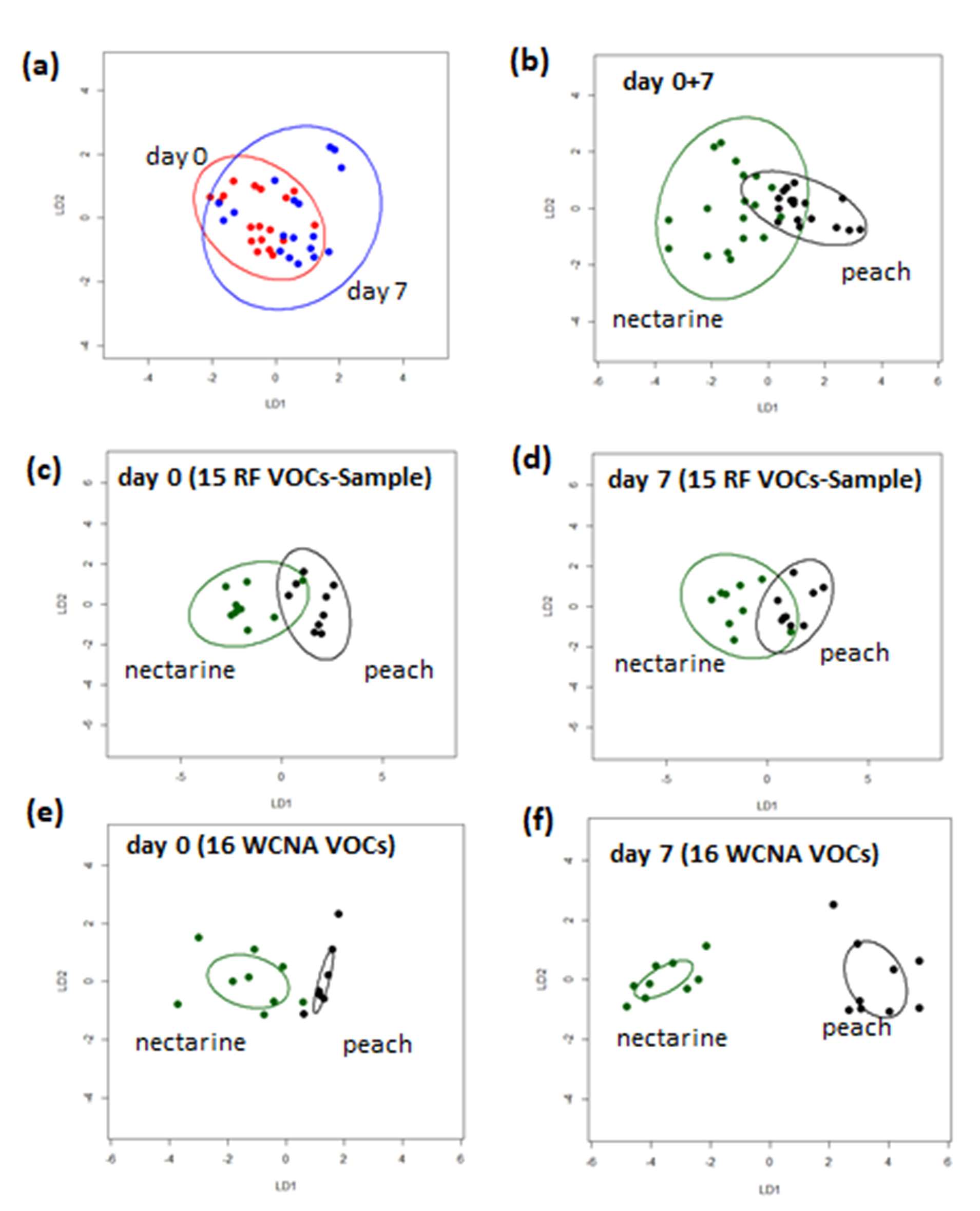

Supplement: Supplementary file 9 — Supplementary Information 9. [file 41598_2020_75322_MOESM9_ESM.tif]
